# A simultaneous journal / wiki publication and dissemination of a new species description: *Neobidessodes darwiniensis* sp. n. from northern Australia (Coleoptera, Dytiscidae, Bidessini)

**DOI:** 10.3897/zookeys.79.803

**Published:** 2011-02-03

**Authors:** Lars Hendrich, Michael Balke

**Affiliations:** 1 Zoologische Staatssammlung, Münchhausenstrasse 21, D-81247 München, Germany; 2 GeoBio Center, Ludwigs-Maximilians-Universität, München, Germany

**Keywords:** Wiki, species ID, online species pages, *cox1*, sequence data, DNA barcoding, molecular biodiversity assessment

## Abstract

Here, we describe a new Australian species in journal format and simultaneously open the description in a wiki format on the www.species-id.net. The wiki format will always link to the fixed original journal description of the taxon, however it permits future edits and additions to species'  taxonomy and biology. The diving beetle Neobidessodes darwiniensis **sp. n.** (Coleoptera: Dytiscidae, Bidessini) is described based on a single female, collected in a rest pool of the Harriet Creek in the Darwin Area, Northern Territory. Within Neobidessodes the new species is well characterized by its elongate oval body with rounded sides, short and stout segments of antennae, length of body and dorsal surface coloration. In addition to external morphology, we used mitochondrial *cox1* sequence data to support generic assignment and to delineate the new species from other Australian Bidessini including all other known Neobidessodes. Illustrations based on digital images are provided here and as online resources. A modified key is provided. Altogether ten species of the genus are now known worldwide, nine from Australia and one from New Guinea.

## Introduction

Many approaches and initiatives to “accelerate” the descriptive taxonomic process have recently been proposed or partially implemented. We suggest that the wikimedia engine provides one of the most powerful tools for routine taxonomic work, with wikipedia providing generic data and wikispecies a taxonomic backbone, i.e. the tree of life (see Page 2010). Here, we test an approach where we publish a new species in open-access journal format, and at the same time upload the data to a purpose-buildt wiki, the species ID site, flanked by a wikispecies entry which *de facto* serves as a "shop window".

The epigean species of the Australasian genus Neobidessodes Hendrich & Balke, 2009 were recently treated in a comprehensive systematic revision, including morphological and molecular data (Hendrich et al. 2009). Two new species, one from Australia and one from New Guinea, were described. Larvae of the genus were described in Michat et al. (2010). In northern Australia, Neobidessodes are among the most common and widespread diving beetles occurring in rest pools of intermittent streams during the dry season. Despite the fact that the first author studied more than 6000 specimens from his own samples and numerous museum collections (Hendrich et al. 2009), the new species described in this publication is known just from the female holotype. The single specimen was until recently overlooked in a vial, including numerous Neobidessodes mjobergi (Zimmermann 1922) and Hydroglpyhus godeffroyi (Sharp 1882) collected in August 2006, on the way from Pine Creek to the Kakadu National Park.

Combining morphology and mitochondrial DNA sequence data we describe the new species and provide a modified key for all epigean species of the genus. The DNA sequence data and a high resolution digital image of the beetle habitus, coloration and sculpture are made available online for faster dissemination of taxonomic knowledge. Links are provided below.

## Material and methods

### Material.

This study is based on the examination of 26 specimens, the holotype of our new species and specimens of Neobidessodes bilita (Watts, 1978) and Neobidessodes mjobergi,deposited in CLH, SAMA and ZSM.

Neobiodessodes bilita (Watts, 1978): Australia, New South Wales. 12 exs., S NSW, 6.5 km SW Eden, Towamba Road 2 km N Nullica, 556 m, 16.XI.2006, 37.04.412S 149.51.200E, L. & E. Hendrich leg. (NSW 111), two specimens with “DNA M.Balke 1900”, “DNA M.Balke 1901” [green printed labels] (CLH, ZSM).

Neobidessodes mjobergi (Zimmermann, 1922): Australia, Northern Territory. 13 exs., Manton Dam Recreation Area, 46 km S Darwin, 35 m, 19.VIII.2006, 12.50.270S 131.08.050E, L. & E. Hendrich leg. (NT 1), one specimen with “DNA M.Balke 1656” [green printed label] (CLH, ZSM).

### Descriptions.

Beetles were studied with a Leica MZ 12.5 dissecting scope at 10–100x. Habitus photos of beetles were made by Alexander Riedel (Karlsruhe, Germany) and by the authors. Image stacks were aligned and assembled with the computer software Helicon Focus 4.77TM. Abbreviations used in the text are: TL (total length), TL-H (total length without head), and MW (maximum width). Label data of type material are cited in quotation marks.

### DNA sequencing and data analysis.

We extracted DNA from the alcohol preserved female holotype after removal of the abdomen, using the Qiagen Dneasy tissue kit. We ran a PCR with Bioline Mago Taq at 94° for 2 min, 40 cycles of 94° for 30 s, 47° for 30 s and 72° for 60 s, and a final extension of 72° for 10 min, using primers for the 3’ end of cytochrome c oxidase 1 (*cox1*) Jerry (F: 5’- CAA CAT TTA TTT TGA TTT TTT GG -3’) and Pat (R: 5’- TCC AAT GCA CTA ATC TGC CAT ATT A -3’) (Simon et al. 1994).

This cox1 fragment is our standard “DNA barcoding” fragment for Dytiscidae, a short fragment of DNA used for preliminary species identification and study of population-level processes (see e.g. Hendrich and Balke 2009 and Hendrich et al. 2009). We used the cox1 fragment sequenced for the female holotype to obtain quantitative data for species recognition which we here suggest especially useful as we had no male specimens for study of male genital structures. The sequence was added to our database of Australian Dytiscidae (Hendrich et al. 2010), containing around 70% of the Australian fauna, including all Neobidessodes species (Hendrich et al. 2009). We ran neighbour joining analysis in PAUP* (Swofford 2002) using HKY85 as well as uncorrected p-distances. The Species Identifier module of Taxon DNA software was used to study sequence divergence in the dataset (Meier et al. 2006).

### Codens

CLHCollection Lars Hendrich, Berlin, Germany; property of the Naturhistorisches Museum Wien, Austria

SAMASouth Australian Museum, Adelaide, South Australia, Australia

ZSMZoologische Staatssammlung München, Munich, Germany

## Results and discussion

### DNA Sequencing

We obtained 450 bp 3’ *cox* 1 sequence (GenBank accession # FR733592). Ran against our 1400+ Australian *cox1* sequence database, we find minimum uncorrected p-distances in SpeciesIdentifier of 10.15% (Limbodessus jundeensis Watts and Humphreys, 2003), followed by Neobidessodes samkrisi Hendrich & Balke, 2009, Neobidessodes thoracicus Hendrich & Balke, 2009 and Neobidessodes bilita (Watts, 1978) (10.37–10.39%) and e.g. Copelatus tenebrosus Régimbart, 1880 (10.59%). The neighbour joining analysis in PAUP* placed Neobidessodes darwiniensis sp. n. as the sister of all other Neobidessodes. This is not necessarily the correct phylogenetic position of Neobidessodes darwiniensis, but indicates that the female studied here does not belong to any other known Neobidessodes, nor to any other species in our database.

## Taxonomy

Neobidessodes is a genus with 10 species distributed in Australia (9 species) and New Guinea (1 species). All but two [the stygobitic Neobidessodes limestonensis (Watts and Humphreys, 2003) and Neobidessodes gutteridgei (Watts & Humphreys, 2003)] species have a more or less contrasting black/yellow surface. The basic pattern of these species includes various yellow or reddish spots. The median lobes are simple and very elongate, in ventral view strongly tapered or rounded at tip. The size of the species varies from 1.95 to 3.85 mm (see also Hendrich et al. 2009).

The new species was placed in Neobidessodes because of the following combination of characters: 1) body elongate oval; 2) basal pronotal striae sharply incised, not connected by a transverse groove; 3) elytra lacking basal striae and sutural striae; 4) epipleura lacking transverse carina; 5) head lacking cervical line and its foremargin not bordered; 6) prosternal process broad, distinctly excavated and marginated; 7) inner margin of both metacoxal wings strongly ridged; 8) hind margin of abdominal ventrites 3–5 without row of minor irregular dentate processes.

### Checklist of Neobidessodes species

NSW= New South Wales; NT= Northern Territory; QLD= Queensland; VIC= Victoria; WA= Western Australia; N= northern; S= southern.

#### Australia – epigean

Neobidessodes bilita (Watts, 1978)	S QLD, NSW, VIC

Neobidessodes darwiniensis Hendrich & Balke, sp. n.	NT

Neobidessodes denticulatus (Sharp, 1882)	N WA, NT, QLD, NSW

Neobidessodes flavosignatus (Zimmermann, 1922)	N WA, NT, N QLD

Neobidessodes grossus (Zimmermann, 1922)	N WA, NT, N QLD

Neobidessodes mjobergi (Zimmermann, 1922)	N WA, NT, N QLD

Neobidessodes thoracicus Hendrich & Balke, 2009	N WA, NT, N QLD

#### Australia – stygobitic

Neobidessodes gutteridgei (Watts & Humphreys, 2003)	WA (Yilgarn)

Neobidessodes limestonensis (Watts & Humphreys, 2003)	WA (Yilgarn)

#### New Guinea – epigean

Neobidessodes samkrisi Hendrich & Balke, 2009	West Papua, Merauke, Indonesia

### Neobidessodes darwiniensis sp. n.

urn:lsid:zoobank.org:act:CAD876B9-A027-458D-88D1-47E4A239FFA7

http://www.species-id.net/wiki/Neobidessodes_darwiniensis

Figs 1 5, 6, 7 

#### Type locality.

Rest pool, Harriet Creek at Kakadu Highway, 11 km NE Pine Creek, Northern Territory, Australia [13°45'04.63"S,  131°53.5531"E].

#### Type material.

**Holotype**: Female, “Australia: NT, Kakadu Hwy, Harriet Creek at Hwy Cross., 156m, 24.VIII.2006, 13.744816S, 131.897483E, L. & E. Hendrich leg. (NT 14)”; “DNA M. Balke 3821” [green printed label]; “HOLOTYPE Neobidessodes darwiniensis sp. n. Hendrich & Balke 2010” [red printed label] (SAMA).

**Figure:**
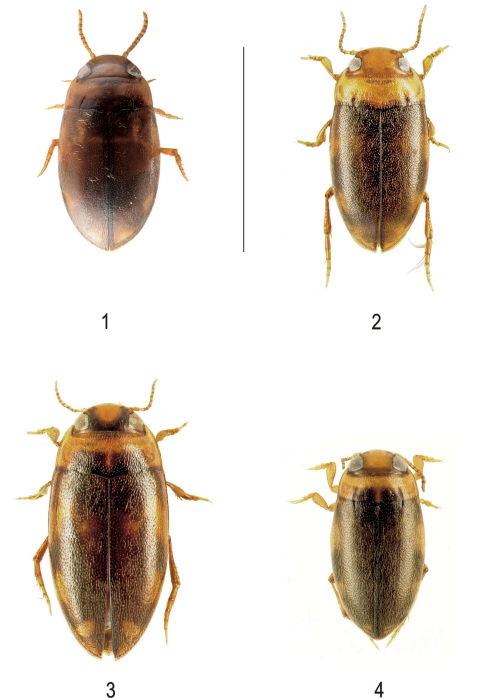
**Figures 1–4.** Habitus of **1** Neobidessodes darwiniensis sp. n. (holotype, female) **2** Neobidessodes bilita (female) **3** Neobidessodes mjobergi and **4** Hydroglyphus godeffroyi (scale bar = 2.0 mm) (Photos: M. Balke, A. Riedel).

#### Description.

Measurements. TL = 1.95 mm, TL-H = 1.8 mm; MW = 1.0 mm.

#### Colour.

Antennae, palpi, head and most parts of pronotum reddish-brown, posterior angles of head, near eyes and base of pronotum in middle broadly dark brown. Elytron dark brown with some small vague yellow spots subbasally and subapically (Fig. 1). Ventral side, including legs and epipleura, reddish-brown, prosternal process and metacoxal plates somewhat darkened.

#### Sculpture and structure.

Elongate oval, sides well rounded. Maximum width at apical third of body. Segments of antennae short and stout. Head with relatively coarse punctures and strong microreticulation. Pronotum and elytron with rather dense, medium-sized punctures and weak to moderate microreticulation, finely pubescent. Pronotal striae deep and well marked, length almost 1/2 of that of pronotum, strongly incurved converging anteriad (Fig. 1). Elytra lacking basal and sutural striae. Underside with a few moderately large weak punctures at sides, midline of metaventrite with moderately dense smaller punctures. Metacoxal lines raised, well separated, weakly diverging anteriorly.

#### Male.

Unknown.

#### Female.

Pro- and mesotarsi simple. Inner edge of mesotibia nearly straight.

#### Affinities - DNA Sequence Data.

The 3’ *cox1* sequence available at http://www.ncbi.nlm.nih.gov/nuccore/FR733592. 1 indicates that the new species is rather distinctive, the closest uncorrected p-distances in our database were other Neobidessodes species (*c*. 10.37%) and Limbodessus jundeensis (10.15%).

#### Morphology.

The smallest species of the genus. On first view, the new species resembles in size and colour the common Hydroglyphus godeffroyi (Fig. 4) distributed all over northern Australia and New Caledonia, and can be easily overlooked in the field. When recognized as a Neobidessodes the new species is similar to Neobidessodes mjobergi (Fig. 3) in coloration and to Neobidessodes bilita (Watts, 1978) (Fig. 2) in size. From Neobidessodes mjobergi it can be separated by its more broadly oval body, the much smaller size (Neobidessodes mjobergi 2.55-2.65 mm) and unicolourus head, and from Neobidessodes bilita by the darker dorsal surface, the short and stout segments of antennae, the rounded, broadly oval body, and the unflanged subapical part of the elytra (Figs 1, 2). Furthermore, Neobidessodes bilita is a strictly south-eastern species with a disjunct distribution from southern Queensland to Victoria (Hendrich et al. 2009).

#### Etymology.

Named after the Darwin area in the Northern Territory; the specific epithet is an adjective in the nominative singular.

#### Distribution.

Only known from the type locality at Harriet Creek, 11 km NE Pine Creek but probably more widespread in the Northern Territory (Fig. 5).

**Figure:**
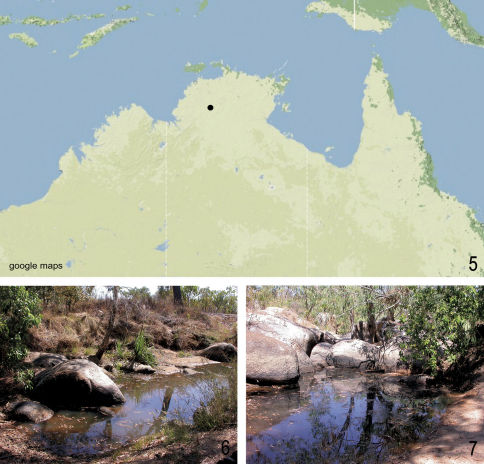
**Figures 5–7. 5** Distribution of Neobidessodes darwiniensis sp. n. in Northern Australia. **6–7** Habitat of Neobidessodes darwiniensis sp. n., Neobidessodes grossus, Neobidessodes  mjobergi and Neobidessodes thoracicus, Northern Territory Kakadu Hwy, Harriet Creek at Hwy Crossing (NT 14) (Photos: L. Hendrich).

#### Habitat.

The single specimen was collected in one of the rest pools of a rocky creek, with gloomy water and at least partly shaded by smaller gum trees. The bottom consisted of coarse sand with a thick layer of unrotten leaves and twigs, no submerged or emergent vegetation visible (Figs 6, 7).

Neobidessodes darwiniensis sp. n. was associated with the dytiscids Clypeodytes larsoni Hendrich & Wang, 2006, Hydroglyphus daemeli (Sharp, 1882), Hydroglyphus godeffroyi, Hydroglyphus grammopterus (Zimmermann, 1928), Hyphydrus contiguus Wehncke, 1877, Hydroglyphus lyratus Swartz, 1808, Laccophilus cingulatus Sharp, 1882, Laccophilus sharpi Régimbart, 1889, Laccophilus walkeri J. Balfour-Browne, 1939, Limbodessus compactus (Clark, 1862), Neobidessodes grossus (Zimmermann, 1922), Neobidessodes mjobergi, Neobidessodes thoracicus Hendrich & Balke, 2009, Sternopriscus alligatorensis Hendrich & Watts, 2004, Sternopriscus aquilonaris Hendrich & Watts, 2004, Tiporus centralis (Watts, 1978), Tiporus guiliani (Watts, 1978) and Tiporus undecimmaculatus (Clark, 1862).

#### Remarks.

Despite the fact that thousands of Neobidessodes were collected on three field trips to the Northern Territory and the Kimberley region, surprisingly only one specimen of Neobidessodes darwiniensis sp. n. appeared. Most of the expeditions took place during the dry period, between June and October, when most of the other species of the genus dominate the remaining rest pools and swamps. We assume the new species is more common in or just after the rainy season, from November to April, as was observed for Neobidessodes grossus (Hendrich et al. 2009).

### The key to epigean species of Neobidessodes in Hendrich et al. (2009) should be modified as follows:

**Table:** 

1	Length > 3.7 mm. Elytron with a subapical lateral flange, pronotal striae very weak, N WA, NT, N QLD	Neobidessodes grossus
–	Length < 3.7 mm	2
2	Elytron with a subapical lateral tooth, pronotal striae well marked, WA, NT, QLD, N NSW	Neobidessodes denticulatus
–	Elytron lacking lateral tooth, pronotal striae present or absent	3
3	Pronotal striae absent	4
–	Pronotal striae present	5
4	Length 2.55–2.65 mm, outline of junction of pronotum and elytra smooth, sides of pronotum evenly curved, maximum width at posterior angles, dorsal colour pattern usually diffuse, N WA, NT, N QLD	Neobidessodes mjobergi
–	Length 2.75–2.9 mm, outline of junction of pronotum and elytra slightly sinuate, maximum width of pronotum somewhat before base. Dorsal colour pattern strongly varying, when present, usually well marked. In some specimens pronotum yellow, in others pronotum and elytra all black, N WA, NT, N QLD	Neobidessodes thoracicus
5	Dorsal colour pattern diffuse. Pronotal striae well marked and long (1/4 to 1/3 of length of pronotum)	6
–	Contrasting yellowish markings on black elytra. Pronotal striae only slightly marked and short (maximum 1/4 of length of pronotum)	7
6	Body elongate oval. Pronotum as broad as elytra, outline of junction of pronotum and elytra slightly sinuate, maximum width of pronotum somewhat before base (Fig. 2). Males with mesotibia curved, length 2.2–2.25 mm, VIC, NSW, S QLD	Neobidessodes bilita
–	Body broader oval. Pronotum narrower than elytra, outline of junction of pronotum and elytra smooth, sides of pronotum evenly curved, maximum width at posterior angles (Fig. 1). Male unknown, length 1.95 mm, smallest species of the genus, NT	Neobidessodes darwiniensis sp. n.
7	Males with mesotarsus straight. Pronotal striae well marked but short, small species, length 2.0 mm, West Papua, Indonesia	Neobidessodes samkrisi
–	Males with mesotarsus straight. Pronotal striae extremely weak and faint, larger species, length 2.35–2.65 mm, N WA, NT, N QLD	Neobidessodes flavosignatus

## Supplementary Material

XML Treatment for Neobidessodes darwiniensis
